# An Appraisal of Nonmicrobial Biostimulants’ Impact on the Productivity and Mineral Content of Wild Rocket (*Diplotaxis tenuifolia* (L.) DC.) Cultivated under Organic Conditions

**DOI:** 10.3390/plants13101326

**Published:** 2024-05-11

**Authors:** Michele Ciriello, Emanuela Campana, Giuseppe Colla, Youssef Rouphael

**Affiliations:** 1 Department of Agricultural Sciences, University of Naples Federico II, 80055 Portici, Italy; michele.ciriello@unina.it (M.C.); emanuela.campana@unina.it (E.C.); 2 Department of Agriculture and Forest Sciences, University of Tuscia, 01100 Viterbo, Italy; giucolla@unina.it

**Keywords:** sustainable horticulture, nutritional status, nitrate, seaweed extract, vegetal-based protein hydrolysate, plant extract, yield gap

## Abstract

Modern agriculture urgently requires viable alternatives to synthetic chemical substances, such as pesticides and fertilizers, to comply with new and stringent international regulations and meet the growing demands of consumers who prefer chemical-free food. Consequently, organic agriculture has garnered increasing interest over time. To compensate for yield reduction resulting from opting out of the use mineral fertilizers, research has focused on the use of biostimulants to sustain the productivity of horticultural crops. To this end, a greenhouse experiment was conducted to assess the effects of three nonmicrobial biostimulants (a plant extract, vegetable protein hydrolysate, and a seaweed extract) and an untreated control on the production and mineral content of wild rocket (*Diplotaxis tenuifolia* (L.) DC.) cultivated under organic conditions and harvested three times during the growth cycle. In general, the nitrate content, which defines the commercial quality of wild rocket, was not influenced by the application of biostimulants. At each harvest, the application of biostimulants resulted in improved production performance, although this was not always accompanied by an increase in mineral content. Specifically, the best results were obtained with the use of plant-derived protein hydrolysate and plant extract, which led to an improvement in total yield of 32.1% and 27.2%, respectively compared to that of control plants. These results reconfirm that biostimulants represent a valid and indispensable tool for organic growers.

## 1. Introduction

In today’s society, people demonstrate a growing propensity to stay informed about environmental issues, probably due to concerns about the possible adverse effects of climate change on human health. This concern has led consumers to adopt healthy eating habits [1,2], rich in vegetables with high antioxidant content, capable of preventing or reducing health problems [3,4]. However, in modern society, people often find themselves under pressure due to the hectic pace of their days. Consequently, time has become a precious commodity and many people seek foods that are not only healthy but also quick to prepare and consume, such as leafy vegetables, which are basic components of ready-made salads [5,6]. Therefore, the production of small leafy vegetables such as arugula, spinach, and lettuce is increasing, given their ease of handling by consumers [7]. To adequately respond to market demand throughout the year, successive harvests are carried out [8], allowing for both an increase in overall yield compared to that of single harvests and an improvement in resource and labor efficiency [9].

In Europe, Italy currently leads the production of leafy vegetables, with approximately 15,000 hectares under protected cultivation, spread between the southern region of Campania and the northern regions of Lombardy and Veneto [10]. Among the various leafy vegetables available on the Italian market, wild rocket (*Diplotaxis tenuifolia* (L.) D.C.) is currently of particular interest due to its nutritional properties and its use in ready-made salads [11]. This plant, which belongs to the *Brassicaceae* family, is rich in phytochemical compounds of high nutritional value, such as polyphenols, glucosinolates, and carotenoids [12,13]. However, the quality of wild rocket is often compromised by its tendency to accumulate high levels of nitrates, which are precursors of carcinogenic compounds [14,15]. For this reason, the nutritional and commercial quality of rocket and other leafy vegetables of the *Brassicaceae, Chenopodiaceae*, and *Asteraceae* families is regulated by the European Commission (EC) through Regulations No. 1881/2006 and No. 1258/2011, which establish maximum levels of nitrates according to species and growing season.

Furthermore, consumers’ growing concern about environmental and health issues drives them not only to seek healthy and convenient foods, but also products produced sustainably and through low-impact cultivation techniques [16]. Therefore, modern organic agriculture is subject to strict prohibitions of the use of synthetic pesticides and fertilizers. Currently practiced in 187 countries, organic agriculture is more prevalent in Europe and Oceania, with countries such as Australia, Spain, and Argentina leading in terms of cultivated land [17,18]. However, its reduced yield, caused by the limited availability of key nutrients such as nitrogen and phosphorus and the increase in biotic pressure, presents significant challenges [19,20]. However, farmers are interested in maintaining quality and yields comparable to those of conventional agriculture, which often leads to increased land use, thus reducing the environmental benefits of organic practices [21].

To address this challenge, researchers and farmers are exploring various sustainable strategies to increase yields in organic agriculture [22]. Among these, the use of biostimulants has been promising, especially those of natural plant origin, which can be obtained from the transformation of agro-food waste [23,24,25]. These bioproducts, rich in endogenous hormones, free amino acids, peptides, and phenols [22,26], can improve crop performance in terms of yield, resistance to environmental stress, and quality [27,28]. However, the efficacy of biostimulants varies according to species and cultivar, environmental conditions, timing, and application methods, which makes standardizing their use challenging [29]. However, the strong interest of the scientific community and growers in the use of biostimulants, from both the perspective of a circular economy and in the context of sustainable agricultural practices that can be implemented in organic farming, has led to more and more studies setting themselves the goal of validating scientific protocols that clarify the mechanisms of action of the different biostimulants [30,31]. The different environmental conditions in the super-intensive production cycles typical of wild rocket cultivation, as well as the mechanical stress induced by cutting, can influence and vary the positive effects of biostimulants, making interpretations by farmers complex. In this regard, the aim of our work is to compare and evaluate the effects of three different types of nonmicrobial biostimulants (plant extract, vegetable protein hydrolysate, and seaweed extract) on the organic production of wild rocket (*Diplotaxis tenuifolia* (L.) D.C.) subject to three successive harvests.

## 2. Results

### 2.1. Marketable Yield, Biometric Components, and Climatic Trends inside the Greenhouse

As shown in Table 1, the mean values of temperature and PPFD, relative to the time slot 7:00 a.m–5 p.m, increased over the course of the harvests with the highest values (15.70 and 600.53) recorded in Harvest III. The opposite trend was observed for the mean relative humidity values where values ranged from 74.96% (Harvest I) to 65.92% (Harvest III).

The results of the one-way ANOVA for all the biometric and yield parameters analyzed are shown in Table 2. Compared to the that of the control, the application of biostimulants (PE, PH, and SWE) resulted in a significant increase in the yield for each harvest (Harvest I, Harvest II, and Harvest III). Specifically, the use of plant-origin protein hydrolysate (PH) led to increases of 25.0%, 43.2%, and 20.9%, respectively, in Harvest I, Harvest II, and Harvest III compared to untreated plants.

**Figure 1:**
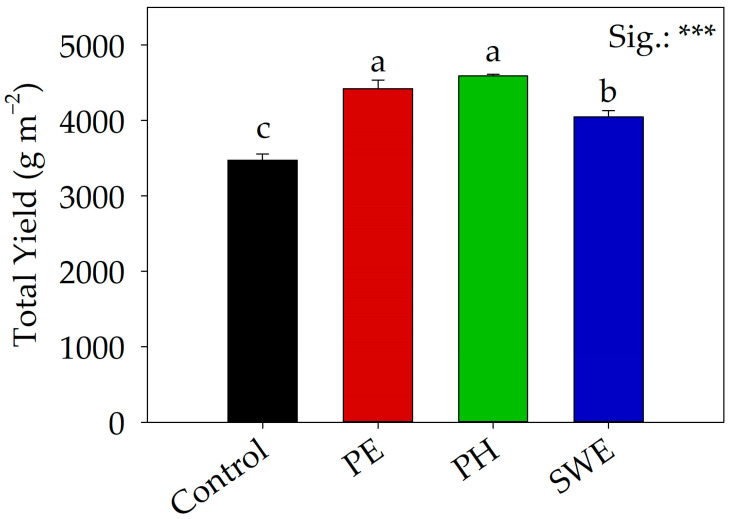
Effect of foliar applications of plant extract (PE), vegetable protein hydrolysate (PH), and seaweed extract (SWE) on the total marketable yield of greenhouse-grown wild rocket. Different letters indicate significant mean differences according to Tukey HSD test (*p* = 0.05). *** denotes significant effects at *p* ≤ 0.001. Data are mean values ± standard deviation, *n* = 3.

The same trend was observed for total yield (Figure 1), where the application of plant extract (PE), PH, and seaweed extract (SWE) resulted in increases of 27.2%, 32.1%, and 16.5%, respectively, compared to the those of the control. However, the highest values were obtained from plants treated with PH and PE. In contrast, dry matter (%) was not significantly influenced by biostimulant treatment. Significant differences in dry biomass were observed only in Harvest I and Harvest II. Specifically, the use of PE and PH in Harvest I resulted, on average, with an increase in the latter of 20.6%. On the other hand, in the second harvest, the use of PH resulted in an increase in dry biomass of 30.3% compared to that of the untreated control.

### 2.2. Mineral Content of Wild Rocket Leaves

The results of the application of biostimulants in the first harvest, as shown in Table 3, revealed significant differences only in leaf nitrate, magnesium (Mg), and sulfur (S). Specifically, nitrate ranged from 7386.43 to 6528.70 mg kg^−1^ fw, respectively, in plants treated with PH and SWE. Compared with that of the control plants, the application of the latter (PH and SWE) resulted in a significant increase in Mg content. With regard to S content, the use of SWE resulted in a significant increase of 15.2% compared to that of the control plants. For potassium (K), the most abundant macro-element, the recorded values ranged from 45.67 g kg^−1^ dw (control) to 52.63 g kg^−1^ dw (PH). For calcium (Ca) and phosphorus (P), the ranges were 20.52–24.58 g kg−1 dw and 1.94–2.18 g kg^−1^ dw, respectively.

Regarding the mineral profile of the plants harvested at the second cut (Table 3), the use of biostimulants showed significant differences in nitrate, K, Mg, and P. Regardless of the origin of the biostimulant (PE, PH and SWE), the nitrate content in the treated plants did not differ significantly from that recorded in the control plants (5522.125 mg kg^−1^ fw). On the contrary, the application of PE resulted in a significant increase in K and Mg of 15.2% and 22.7%, respectively, compared to untreated plants. PH-treated plants had 13.9% higher P content than control plants. For Ca and S, the ranges were 16.70–20.34 g kg^−1^ dw and 7.11–8.13 g kg^−1^ dw, respectively.

As reported in Table 3, the application of different biostimulants products did not lead to significant differences for any quantified parameter. Nevertheless, nitrate ranged from 4421.55 to 4801.12 mg kg^−1^ fw, respectively, in the plants treated with PE and PH.

## 3. Discussion

As with most baby-leaf crops, successive harvesting is a widely adopted agronomic practice in wild rocket cultivation. As Petropoulos et al. [9] emphasizes, this approach has the potential to significantly improve overall yield compared to that of singular harvests. However, as extensively noted in the existing literature, fluctuations in climatic parameters (such as temperature, light intensity, photoperiod, and relative humidity) throughout the growth cycle pose challenges in deciphering the cause-and-effect dynamics between harvesting and plant productivity. The variations in climatic parameters recorded in our experiment (Table 1) substantiate the varying durations of the vegetative cycle between different harvests. Consequently, for each harvest, our focus remained exclusively on the actions exerted by the different nonmicrobial biostimulants tested (PE, PH, and SWE).

Regardless of the application of biostimulants, the total yield (Figure 1) was lower compared to that of the recent findings reported by Caruso et al. [32], being mainly attributed to our data originating from organic cultivation, which is inherently less productive than conventional methods. Furthermore, references to the literature indicate the potential for wild rocket to be harvested up to five times, especially during the winter–spring period, while our study reported total production for only three harvests [16,30]. As shown in Figure 1, the application of biostimulants, regardless of their origin, significantly increased total yield, corroborating the findings of Caruso et al. [33] for rocket and Hassan et al. [34], Mannino et al. [35], Carillo et al. [36], and Admane et al. [37] for various crops such as tomato (*Solanum lycopersicum* L.), cucumber (*Cucumis sativus* L.), spinach (*Spinacia oleracea* L.), and lettuce (*Lactuca sativa* L.). Given the organic context of the experiment, the increase in total yield (+25.2% on average) is of greater significance. This heightened production underscores the crucial role of biostimulant products in nonconventional farming, susceptible as it is to the biotic and abiotic stresses characteristic of organic agriculture.

Ertani et al. [38] and Fan et al. [39] argue that the observed beneficial effects of the application of different biostimulants are attributable to the direct and indirect activation of physiological and/or molecular mechanisms, such as the elicitation of hormone activity, the improvement in nutritional status, and the positive modification of the root system. Despite the marked chemical disparities among the biostimulants used, all contributed positively to rocket growth and production. Although biostimulants derived from macroalga extracts generally have high phytohormone levels [40], plants treated with SWE exhibited lower yields compared to those treated with PE and PH, probably due to the brevity of individual crop cycles (maximum 46 days), preventing the beneficial effects of phytohormones. Consistent with the findings of Baltazar et al. [31] and Giordano et al. [12] in similar rocket experiments, plant-based biostimulants (PE and PH) did not show significant differences, indicating a shared mode of action. This may be partly attributed to the presence of peptides and amino acids in both formulations, which play pivotal physiological roles in plant metabolism, especially in the carbon and nitrogen cycles. However, the total nitrogen content did not show significant differences (Table 2 and Table 3), suggesting that the growth effects do not arise from the absorption of new nitrogen compounds, but rather from the more efficient mobilization and utilization of existing nitrogen reserves by plants.

As reported in Table 2, fresh yield was positively influenced by biostimulant application in all harvests. Regardless of the type of biostimulants, production increased by 20.8%, 31.2%, and 19.9% in the first, second, and third harvests, respectively, compared to in the control conditions. As suggested by Rouphael et al. [41], these positive effects could be related to the intrinsic hormonal action of biostimulants, which, by stimulating root growth, facilitate the absorption, translocation, and transport of nutrients. Although the literature reports often indicate an increased accumulation of key minerals (such as K, Mg, P and Ca) after biostimulant application [42,43], the mineral profile analysis reported in Table 3 partially supports these hypotheses. In particular, in the first harvest, the application of PH and SWE significantly increased Mg content, crucial for the regulation of cellular turgor and photosynthetic processes as a central component of chlorophyll [44]. A similar result was observed in the second harvest for plants treated with PH and PE. However, PE use also increased the K content, the most abundant macroelement in rocket tissues [10]. Despite the role of potassium in the regulation of muscle and myocardial fiber cell excitability in humans [45], the nutritional value of rocket is inversely correlated with nitrate content. Nitrate is considered an antinutritional factor as a result of its indirect implications on human health [14,46]. Its bioaccumulation is a complex characteristic influenced by numerous external and internal factors. For this reason, increasing attention has been paid in recent years to the study of different agronomic practices and how they can contribute to reducing this antinutrient content.

With respect to biostimulants, the literature reports conflicting results on their impact on the nitrate content, which appears to depend on the cropping system and the specific biostimulant product used [47]. Although Sifola et al. [48] and Bulgari et al. [49] reported an increase and a reduction in nitrate content, respectively, after biostimulant application, no differences were observed between biostimulant treatments and their respective controls in our experiment. It should be noted that in this study, the nitrate content in the first harvest for both control plants (7014.04 mg kg^−1^ FW) and those treated with PH (7386.43 mg kg^−1^ FW) exceeded the maximum limits set by the European Union for rocket commercialization (EU Regulation No. 1258/2011 of 2 December 2011). These results are unsurprising, as nitrate values exceeding 9000 mg kg^−1^ FW have been reported in rocket in Italy [50]. Regardless of the application of the biostimulant, the nitrate content in the rocket leaves showed a decreasing trend after the first harvest. This observed trend may be attributed to an increase in air temperature and light intensity during the crop cycle (Table 3). As highlighted by Bonasia et al. [51] and Weightman et al. [16], environmental conditions, together with genotype, directly regulate nitrate reductase (NR) activity. This, in combination with nitrite reductase (NiR) and other enzymes, plays the role of nitrate assimilation and regulation [52]. Specifically, NR reduces nitrate to nitrite, which in turn is reduced to ammonium by NiR. However, the gene expression of NR is bound by high light intensity and high temperatures [53].

Improved light and temperature conditions have a positive impact on not only the nitrate content but also the duration of growth cycle. Specifically, independently of the biostimulant treatment applied, the average value of nitrate accumulated in the three different harvests is found to decrease gradually, with an average nitrate value of 6918.15 mg Kg^−1^ fw in Harvest I, 5550.05 mg Kg^−1^ fw in Harvest II, and 4657.36 mg Kg^−1^ fw in Harvest III. The reduction in mean nitrate values recorded during the three harvests are confirmed by the gradual increase in NR gene expression due to the increase in temperature and light intensity, as shown in Table 1. Regardless of the harvesting factor, Figure 1 shows that the application of the biostimulants evaluated in this experiment significantly increased total yield, once again confirming the positive role of these products.

However, dry weight values did not confirm this trend. Significant differences were observed only in the first and second harvests, showing no variation in the third harvest. Specifically, in the first two harvests, the application of PH increased dry weight by 28.1% and 29.7%, respectively, compared to in the untreated control. In contrast, for all harvests evaluated (Harvest I, II, and III), dry matter content was not influenced by the application of biostimulants, confirming the results obtained by Toscano et al. [54] on Tuscan Black Laciniato Cabbage and on Black Broccoli that were both cultivated as baby leaves.

## 4. Materials and Methods

### 4.1. Growth Conditions and Plant Material

The experiment was carried out during the winter–spring season at the Altamura Agricultural Company, located in Pontecagnano Faiano (Salerno, Italy, 40°38′11.868″ N 14°54′46.008″ E). The main physicochemical characteristics of the soil at the experimental site were clay loam texture (sand 37%, clay 34%, and silt 39%), electrical conductivity (EC) 0.52 uS cm^−1^, pH 8.1, total nitrogen of 0.09%, and organic matter percentage 1.68. Exchangeable potassium and Olsen phosphorus were 191.1 and 105.6 mg kg^−1^, respectively. The cultivation system consisted of three tunnels, each 30 m in length, 4.5 m in peak height, and 7.2 m in width. Each tunnel was covered with a polyethylene film and exposed to natural light. Fertilization, irrigation, and phytopathogen controls were carried out according to organic farming practices. The seeds of the wild rocket cultivar (*Diplotaxis tenuifolia* (L.) DC.) cultivar (Enza Zaden, Enkhuizen, Noord-Holland, Holland) were seeded in the soil at a density of 8 kg ha^−1^. Climatic parameters, such as air temperature, photosynthetically active radiation, and relative humidity, were continuously recorded throughout the experimental period using the WatchDog A150 data logger (Spectrum Technologies Inc., Aurora, IL, USA; precision ±0.6 °C/±3% Temp/RH). The average trend data for temperature, humidity, and light intensity from 7 am. to 5 pm are shown in Table 1. The plants were harvested three times during the growth season from 2 December to 11 March. Each harvest was a production cycle named Harvest I, Harvest II, and Harvest III, which lasted 46, 36, and 18 days, respectively.

### 4.2. Application of Biostimulants and Experimental Design

Three nonmicrobial biostimulants were compared with an untreated control. Biostimulants were a plant extract [hereafter PE (Auxym^®,^ Hello Nature^®^, Rivoli Veronese, Verona, Italy)], a vegetable protein hydrolysate [hereafter PH, Hello Nature^®^], and a seaweed extract [hereafter SWE, Sipcam, Pero, Milan, Italy]. Each biostimulant was applied weekly foliarly at the recommended doses, namely 1.0, 3.0, and 1.5 mL L^−1^, respectively. All biostimulants plus the untreated control were replicated three times for a total of 12 experimental units and arranged in a randomized design. Each experimental unit was 1 m^2^.

### 4.3. Harvest and Biometric Parameters

For each harvest and for each experimental unit, the fresh yield was quantified and expressed as g m^−2^. Subsequently, the harvested plant material was placed in a ventilated oven at 55 °C to determine dry biomass (g m^−2^) and subsequently dry matter (%). Part of the dehydrated and ground material was used to determine the total nitrogen, nitrates, and mineral content.

### 4.4. Determination of Mineral Profile and Total Nitrogen

The mineral content (K, Ca, Mg, P, and S) and the nitrates in the rocket leaves were determined by ion chromatography (ICS3000, Thermo Scientific™ Dionex™, Sunnyvale, CA, USA) coupled with a conductivity detector using Chromeleon™ 6.8 software (Thermo Scientific™ Dionex™, Sunnyvale, CA, USA). Nitrate concentration was expressed as mg kg^−1^ fresh weight (fw), while K, Ca, Mg, P, and S as mg g^−1^ dw. The total nitrogen concentration of the rocket samples was determined using the Kjeldahl methodology.

### 4.5. Statistics

All data were analyzed using IBM SPSS Statistics software (SPSS Inc., Chicago, IL, USA) version 26.0 for Windows 10 and are presented as mean ± standard error, *n* = 3. All mean effects were subjected to a one-way ANOVA analysis. Statistical significance was determined using Tukey’s HSD test at the level *p* = 0.05.

## 5. Conclusions

Consumer interest in safer products free of chemical residues has significantly increased the percentage of organic farmland. However, the lower yields achievable in organic settings clash with the need to increase productivity, making organic farming less sustainable. This critical situation has led research towards identifying sustainable methods and strategies to address and attempt to resolve this issue. The results of our research highlight the key role of biostimulants in organic production contexts. Specifically, the use of the three tested nonmicrobial biostimulants (PE, PH, and SWE) increased the yield of wild rocket (*Diplotaxis tenuifolia* (L.) DC.) without increasing the nitrate content. Based on these promising findings, biostimulants are confirmed as an essential tool to improve organic production. However, to further optimize the use of biostimulants under unfavorable conditions, we need to deepen our understanding of the specific mechanisms employed by these heterogeneous products.

## Figures and Tables

**Table 1:** For each harvest, average trend data for temperature, relative humidity, and light intensity from 7 a.m. to 5 p.m.

	Harvest I	Harvest II	Harvest III
Temperature (°C)	13.99	14.99	15.70
Relative Humidity (%)	74.96	69.06	65.92
PPFD (µmol m^−2^ s^−1^)	252.60	398.50	600.53

**Table 2:** Effect of foliar applications of plant extract (PE), vegetable protein hydrolysate (PH), and seaweed extract (SWE) on marketable yield, dry biomass, and dry matter of three greenhouse-grown wild rocket harvests.

	Control	PE	PH	SWE	*p* Value	sig	F-Value
	**Harvest I**						
Yield (g m^−2^)	994.80 ± 11.29 b	1216.60 ± 33.01 a	1243.92 ± 28.77 a	1145.85 ± 34.68 a	0.001	***	15.34
Dry biomass (g m^−2^)	109.96 ± 1.56 b	132.00 ± 5.51 a	133.22 ± 1.94 a	122.84 ± 4.88 ab	0.010	**	7.65
Dry matter (%)	10.881 ± 0.2	10.87 ± 0.09	10.73 ± 0.17	10.72 ± 0.22	0.862	ns	0.25
	**Harvest II**						
Yield (g m^−2^)	1560.55 ± 36.20 c	2052.89 ± 75.32 ab	2234.35 ± 17.85 a	1856.98 ± 69.83 b	0.000	***	27.31
Dry biomass (g m^−2^)	162.18 ± 8.72 b	197.97 ± 7.36 ab	211.40 ± 0.97 a	179.42 ± 13.65 ab	0.021	*	5.83
Dry matter (%)	9.83 ± 0.32	9.69 ± 0.37	9.40 ± 0.17	9.89 ± 0.40	0.728	ns	0.44
	**Harvest III**						
Yield (g m^−2^)	919.71 ± 35.48 b	1151.70 ± 18.24 a	1112.02 ± 18.61 a	1045.01 ± 33.42 a	0.002	**	13.53
Dry biomass (g m^−2^)	123.81 ± 3.60	115.82 ± 7.19	121.62 ± 1.96	117.00 ± 1.84	0.530	ns	0.79
Dry matter (%)	12.27 ± 0.16	12.00 ± 0.50	11.64 ± 0.19	11.98 ± 0.68	0.789	ns	0.35

ns, *, **, and *** are non-significant or significant at *p* ≤ 0.05, 0.01, and 0.001, respectively. Different letters indicate significant mean differences according to Tukey HSD test (*p* = 0.05). All data are expressed as mean ± standard.

**Table 3:** Effect of foliar applications of plant extract (PE), vegetable protein hydrolysate (PH), and seaweed extract (SWE) on the mineral profile of wild rocket.

	Control	PE	PH	SWE	*p* Value	Sig	F-Value
	**Harvest I**						
Nitrate (mg kg^−1^ fw)	7014.04 ± 134.12 ab	6743.76 ± 94.86 ab	7386.43 ± 245.15 a	6528.70 ± 196.61 b	0.04	*	4.35
Total N (g kg^−1^ dw)	5.55 ± 0.05	5.62 ± 0.04	5.50 ± 0.08	5.65 ± 0.06	0.35	ns	1.27
K (g kg^−1^ dw)	45.67 ± 2.81	50.30 ± 0.89	52.63 ± 3.36	49.30 ± 1.09	0.27	ns	1.58
Mg (g kg^−1^ dw)	4.39 ± 0.11 b	4.72 ± 0.06 ab	4.96 ± 0.05 a	4.80 ± 0.08 a	0.01	**	8.32
Ca (g kg^−1^ dw)	20.52 ± 1.03	22.77 ± 1.80	23.00 ± 1.77	24.58 ± 0.56	0.30	ns	1.44
P (g kg^−1^ dw)	1.94 ± 0.02	1.95 ± 0.08	2.18 ± 0.10	1.95 ± 0.03	0.11	ns	2.84
S (g kg^−1^ dw)	6.35 ± 0.12 b	7.01 ± 0.16 ab	6.34 ± 0.14 b	7.32 ± 0.25 a	0.01	**	7.97
	**Harvest II**						
Nitrate (mg kg^−1^ fw)	5522.12 ± 88.49 ab	5343.25 ± 214.78 b	6095.78 ± 124.64 a	5239.018 ± 118.68 b	0.01	*	6.988
Total N (g kg^−1^ dw)	5.43 ± 0.18	5.39 ± 0.01	5.75 ± 0.23	5.21 ± 0.16	0.24	ns	1.723
K (g kg^−1^ dw)	44.15 ± 1.64 b	50.88 ± 1.93 a	50.01 ± 1.35 ab	46.43 ± 0.70 ab	0.04	*	4.504
Mg (g kg^−1^ dw)	4.01 ± 0.05 b	4.81 ± 0.20 a	4.94 ± 0.13 a	4.53 ± 0.19 ab	0.02	*	6.407
Ca (g kg^−1^ dw)	16.70 ± 0.70	19.25 ± 1.26	20.34 ± 0.86	18.84 ± 0.58	0.10	ns	2.932
P (g kg^−1^ dw)	2.51 ± 0.09 b	2.92 ± 0.05 ab	2.98 ± 0.16 a	2.86 ± 0.04 ab	0.04	*	4.373
S (g kg^−1^ dw)	7.10 ± 0.06	8.13 ± 0.24	7.49 ± 0.38	7.94 ± 0.29	0.10	ns	2.907
	**Harvest III**						
Nitrate (mg kg^−1^ fw)	4605.63 ± 48.73	4421.54 ± 228.61	4801.12 ± 108.39	4498.96 ± 65.06	0.28	ns	1.533
Total N (g kg^−1^ dw)	5.34 ± 0.20	5.14 ± 0.03	5.39 ± 0.06	5.21 ± 0.26	0.72	ns	0.459
K (g kg^−1^ dw)	40.44 ± 1.33	42.82 ± 4.15	47.09 ± 1.63	40.88 ± 1.19	0.31	ns	1.419
Mg (g kg^−1^ dw)	4.05 ± 0.12	4.53 ± 0.10	4.24 ± 0.21	4.50 ± 0.09	0.12	ns	2.678
Ca (g kg^−1^ dw)	13.43 ± 1.08	12.75 ± 0.71	13.12 ± 0.52	12.16 ± 0.56	0.68	ns	0.522
P (g kg^−1^ dw)	1.98 ± 0.08	1.91 ± 0.11	2.12 ± 0.08	2.06 ± 0.09	0.49	ns	0.873
S (g kg^−1^ dw)	7.12 ± 0.40	7.16 ± 0.47	7.29 ± 0.43	7.16 ± 0.21	0.99	ns	0.036

ns, *, and ** are non-significant or significant at *p* ≤ 0.05 and 0.01, respectively. Different letters indicate significant mean differences according to Tukey HSD test (*p* = 0.05). All data are expressed as mean ± standard error, *n* = 3.

## Data Availability

The data are contained within the article.
